# Relativistic finite-difference time-domain analysis of high-speed moving metamaterials

**DOI:** 10.1038/s41598-018-25995-4

**Published:** 2018-05-16

**Authors:** Yan Zhao, Sarawuth Chaimool

**Affiliations:** International School of Engineering, Faculty of Engineering, Chulalongkorn University, Bangkok, 10330 Thailand

## Abstract

In this paper, we apply a relativistic finite-difference time-domain (FDTD) method by using the Lorentz transformation to analyze metamaterials moving at a high speed. As an example, we consider a slab of left-handed metmaterial (LHM) with both relative permittivity and permeability equal to −1. Simulation results show that when the LHM slab moves at a high speed, its electromagnetic responses are drastically different from the static case. Specifically, when the LHM slab moves toward the source, for the case of normal incidence, there exists a special velocity at which fields experience a zero spatial phase delay through the LHM slab; while for the oblique incidence, above a certain velocity fields inside the LHM become evanescent. On the other hand, when the LHM slab moves away from the source, for the case of normal incidence, at the same special velocity the magnitudes of both electric and magnetic fields inside the LHM slab reach their minimum values; for the oblique incidence, the slab functions as a field converter. Besides, the transmitted waves through the LHM slab experience a red-shift (to a lower frequency) and the shift is proportional to the velocity of the LHM slab regardless of the moving direction.

## Introduction

Metamaterials are defined as artificial periodic structures which possess extraordinary and desirable electromagnetic properties that have not been found in naturally occurring materials^1^. Typical applications of metamaterials include superresolution imaging^2,3^, cloaking^4^, perfect wave absorption^5^, and subwavelength image magnification^6,7^ etc. The early development of metamaterials has been focused on the realization of three-dimensional (3-D) structures^8^. However, due to their nature of being lossy, having a narrow bandwidth, and difficulty in fabrication, practical applications of 3-D metamaterials are very limited. For these reasons, more recently, extensive efforts have been put into the analysis and design of metamaterial structures with reduced dimensions, i.e. two-dimensional (2-D) metamaterials, referred to as metasurfaces^9,10^. These thin structures have been shown to possess exceptional abilities to control electromagnetic waves, allow cost-effective fabrications, and may hold promises for future applications in imaging, sensing, and quantum information processing etc.

To date, most of the research works have been conducted for static metamaterial structures, whose material parameters are time invariant and their spatial locations remain unchanged. For dynamic metamaterials, the initial analyses of time-gradient metasurfaces have revealed their interesting properties such as nonreciprocity and frequency tunability^11,12^. Nonetheless, so far the second issue of dynamic metamaterials–when the structure is moving in space–has not been addressed in literature.

In the study of metamaterials, both analytical methods and numerical techniques have been extensively used. The finite-difference time-domain (FDTD) method is especially popular due to its simplicity and capability of handling inhomogeneous, anisotropic, nonlinear, and frequency dispersive materials^13^. In this paper, we develop a numerical model by combining the FDTD method and the Lorentz transformation, for modeling metamaterials moving at a high speed. As an example, we consider a special type of metamaterial–the left-handed metamaterial (LHM) with both relative permittivity and permeability equal to −1. Simulation results show that electromagnetic responses of fast moving LHMs are drastically different from the static case, and many interesting properties can be obtained when the LHM moves at a high speed.

## Methods

In general there are two types of relativistic FDTD methods: applying the relativistic boundary condition (RBC)^14–16^, and applying the Lorentz transformation^17–19^. Each method has its advantages and drawbacks. Specifically, the RBC-based method is applicable for objects moving at any velocity, either constant or varying, however, a mapping of material boundaries to discrete FDTD grid points at every time step is unavoidable due to the movement of the object, which may lead to the numerical instability issue; the Lorentz transformation avoids boundary mapping since the standard FDTD method is used in the moving frame, while the method is only valid for objects moving at a constant velocity.

Both the RBC-FDTD method and the Lorentz-FDTD method have been used to analyze high-speed moving conducting and dielectric objects, and numerical results have shown good agreement with analytical solutions. In this work, due to its simplicity, we extend the Lorentz-FDTD method to take into account the frequency dispersion effects in materials, and then model metamaterials with negative material parameters.

**Figure 1:**
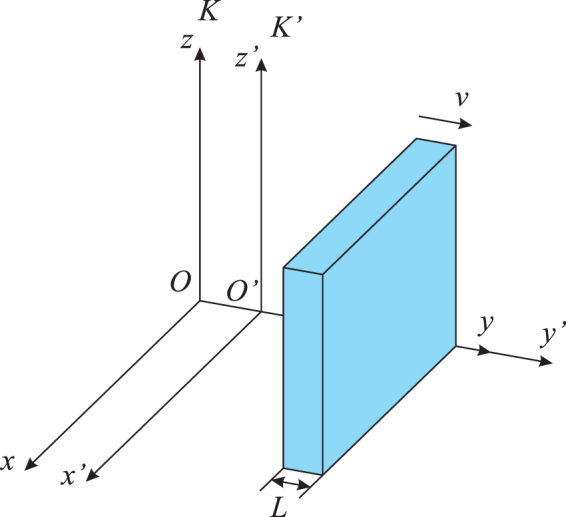
The FDTD simulation domain for modelling metamaterials moving at a velocity **v** = +$v\hat{y}$, containing a *laboratory* frame, *K* which is assumed to be static, and a *rest* frame, *K*′ which moves in the same direction and at the same velocity as the metamaterial.

The Lorentz transformation is a transformation between two coordinate frames that move at a constant velocity relative to each other. Here we consider two coordinate systems, *K* and *K*′ which coincide at time instant *t* = *t*′ = 0. Assuming *K*′ moves at a constant velocity, *v* relative to *K* toward a particular direction. Thus the coordinate system *K*′ is called the *rest* frame, and *K* is referred to as the *laboratory* frame, as illustrated in Fig. 1.

By applying the Lorentz transformation, the electromagnetic fields as well as space and time variables can be transformed between these two systems *K* and *K*′. The transformation formulas for the electromagnetic field components **E**, **H**, **D**, **B** in the general form are expressed by^20^:

1 $${\bf{B}}^{\prime} =\xi ({\bf{B}}-\frac{{\bf{v}}\times {\bf{E}}}{{c}^{2}})-(\xi -\mathrm{1)(}{\bf{B}}\cdot \hat{{\bf{v}}})\hat{{\bf{v}}},$$

2 $${\bf{E}}^{\prime} =\xi ({\bf{E}}+{\bf{v}}\times {\bf{B}})-(\xi -\mathrm{1)(}{\bf{E}}\cdot \hat{{\bf{v}}})\hat{{\bf{v}}},$$

3 $${\bf{D}}^{\prime} =\xi ({\bf{D}}+\frac{{\bf{v}}\times {\bf{H}}}{{c}^{2}})+\mathrm{(1}-\xi )({\bf{D}}\cdot \hat{{\bf{v}}})\hat{{\bf{v}}},$$

4 $${\bf{H}}^{\prime} =\xi ({\bf{H}}-{\bf{v}}\times {\bf{D}})+\mathrm{(1}-\xi )({\bf{H}}\cdot \hat{{\bf{v}}})\hat{{\bf{v}}},$$

where

$$\xi =\frac{1}{\sqrt{1-{v}^{2}/{c}^{2}}}$$

is called the Lorentz factor, *c* is the speed of light in free space, and $\hat{{\bf{v}}}$ is the velocity unit vector. The above transformations allow the calculation of field components in the *rest* frame, *K*′. However in FDTD, in order to analyze field scattering from moving objects in the *laboratory* frame *K*, it is necessary to solve the above equations for the **B**, **E**, **D**, **H** components using their counterparts in the *rest* frame *K*′. Relevant equations are provided in the next section for 1-D and 2-D cases.

The FDTD method was first proposed by Yee in 1966^21^. The method is based on an iterative process which allows to obtain electromagnetic fields throughout the computational domain at a certain time step in terms of fields at previous time steps using a set of updating equations^13^. The typical discretization scheme involves forming a dual-electric-magnetic field grid with electric and magnetic cells spatially and temporally offset from each other. The main advantages of the FDTD method are its simplicity, effectiveness and accuracy as well as the capability of handling frequency dispersive and anisotropic materials. Although the FDTD method has been extensively used to model various types of materials including metamaterials^22–24^, its applications to analyze electromagnetic responses from moving objects are still limited^14–19,25^. To the best of authors’ knowledge, the FDTD method has not been used to analyze high-speed moving metamaterials, which behave considerably different comparing with the static case, as will be shown later in this paper. Besides, based on our proposed method, other types of metamaterials such as metasurfaces^9,10^ when moving at a high speed, can be conveniently analyzed in a straightforward manner.

Due to their frequency dispersive nature, metamaterials can be modelled as an effective medium, and are usually characterized by the Drude dispersion model:

5 $${\varepsilon }_{r}(\omega ^{\prime} )={\mu }_{r}(\omega ^{\prime} )=1-\frac{{\omega }_{p}^{2}}{\omega {^{\prime} }^{2}-j\omega ^{\prime} \gamma },$$

where *ω*′ is the angular frequency in the *rest* frame, and *ω*_*p*_ and *γ* are plasma and collision frequencies, respectively. Here we assume both permittivity and permeability to have the same dispersion form, which is suited for modeling the isotropic LHM with *ε*_*r*_ = *μ*_*r*_ = −1.

In order to model frequency dispersive materials using the FDTD method, the auxiliary differential equation (ADE) method is applied, which is based on the Faraday’s and Ampere’s laws:

6 $$\nabla \times {\bf{E}}{\boldsymbol{^{\prime} }}=-\,\frac{\partial {\bf{B}}{\boldsymbol{^{\prime} }}}{\partial t},$$

7 $$\nabla \times {\bf{H}}^{\prime} =\frac{\partial {\bf{D}}{\boldsymbol{^{\prime} }}}{\partial t},$$

as well as the constitutive relations **D**′ = *ε*_0_*ε*_*r*_**E**′ and **B**′ = *μ*_0_*μ*_*r*_**H**′ where *ε*_0_ and *μ*_0_ are free-space permittivity and permeability, respectively, and *ε*_*r*_ and *μ*_*r*_ are expressed by (5). Note that in the above and following equations in this section, all field components, FDTD cell size, and the discrete time step refer to those in the *rest* frame, since the LHM remains static in that frame, and the ADE dispersive FDTD method is applied.

Equations (6) and (7) can be discretized following a standard procedure^13,21^ which leads to the conventional FDTD updating equations:

8 $${\bf{B}}{^{\prime} }^{n+1}={\bf{B}}{^{\prime} }^{n}-{\rm{\Delta }}t^{\prime} \cdot \tilde{\nabla }\times E{^{\prime} }^{n+\frac{1}{2}},$$

9 $${\bf{D}}{^{\prime} }^{n+1}={\bf{D}}{^{\prime} }^{n}+\Delta t^{\prime} \cdot \tilde{\nabla }\times {{\bf{H}}{\boldsymbol{^{\prime} }}}^{n+\frac{1}{2}},$$

where $\tilde{\nabla }$ is the discrete curl operator, Δ*t*′ is the discrete FDTD time step and *n* is the number of time steps. In addition, auxiliary differential equations have to be taken into account and they can be discretised through the following steps. The constitutive relation between **D**′ and **E**′ reads

10 $$({\omega }^{2}-j\omega ^{\prime} \gamma ){\bf{D}}{\boldsymbol{^{\prime} }}={\varepsilon }_{0}(\omega {^{\prime} }^{2}-j\omega ^{\prime} \gamma -{\omega }_{p}^{2}){\bf{E}}^{\prime} \mathrm{.}$$

Using inverse Fourier transform and the following rules:

$$j\omega ^{\prime} \to \frac{\partial }{\partial t},\,\,\omega {^{\prime} }^{2}\to -\frac{{\partial }^{2}}{\partial {t}^{2}},$$

Equation (10) can be rewritten in the time domain as

11 $$(\frac{{\partial }^{2}}{\partial t{^{\prime} }^{2}}+\frac{\partial }{\partial t^{\prime} }\gamma ){\bf{D}}^{\prime} ={\varepsilon }_{0}(\frac{{\partial }^{2}}{\partial t{^{\prime} }^{2}}+\frac{\partial }{\partial t^{\prime} }\gamma +{\omega }_{p}^{2}){\bf{E}}{\boldsymbol{^{\prime} }}\mathrm{.}$$

The FDTD simulation domain is represented by an equally spaced 3-D grid with periods Δ*x*′, Δ*y*′ and Δ*z*′ along *x*′-, *y*′- and *z*′-directions, respectively. Following a standard discretization procedure^13^, Eq. (11) can be approximated as

12 $$\begin{array}{rcl}\frac{{\bf{D}}{^{\prime} }^{n+1}-2{\bf{D}}{^{\prime} }^{n}+{\bf{D}}{^{\prime} }^{n-1}}{{({\rm{\Delta }}t^{\prime} )}^{2}}+\gamma \frac{{\bf{D}}{^{\prime} }^{n+1}-{\bf{D}}{^{\prime} }^{n-1}}{2{\rm{\Delta }}t^{\prime} } & = & {\varepsilon }_{0}[\frac{{\bf{E}}{^{\prime} }^{n+1}-2{\bf{E}}{^{\prime} }^{n}+{\bf{E}}{^{\prime} }^{n-1}}{{({\rm{\Delta }}t^{\prime} )}^{2}}+\gamma \frac{{\bf{E}}{^{\prime} }^{n+1}-{\bf{E}}{^{\prime} }^{n-1}}{2{\rm{\Delta }}t^{\prime} }\\  &  & +{\omega }_{p}^{2}\frac{{\bf{E}}{^{\prime} }^{n+1}+2{\bf{E}}{^{\prime} }^{n}+{\bf{E}}{^{\prime} }^{n-1}}{4}]\mathrm{.}\end{array}$$

Therefore the updating equation for **E**′ in terms of **E**′ and **D**′ at previous time steps is as follows:

13 $$\begin{array}{rcl}{{\bf{E}}{\boldsymbol{^{\prime} }}}^{n+1} & = & \{[\frac{1}{{\varepsilon }_{0}{({\rm{\Delta }}t^{\prime} )}^{2}}+\frac{\gamma }{2{\varepsilon }_{0}{\rm{\Delta }}t^{\prime} }]{{\bf{D}}{\boldsymbol{^{\prime} }}}^{n+1}-\frac{2}{{\varepsilon }_{0}{({\rm{\Delta }}t^{\prime} )}^{2}}{{\bf{D}}{\boldsymbol{^{\prime} }}}^{n}\\  &  & +[\frac{1}{{\varepsilon }_{0}{({\rm{\Delta }}t^{\prime} )}^{2}}-\frac{\gamma }{2{\varepsilon }_{0}{\rm{\Delta }}t^{\prime} }]{{\bf{D}}{\boldsymbol{^{\prime} }}}^{n-1}+[\frac{2}{{({\rm{\Delta }}t^{\prime} )}^{2}}-\frac{{\omega }_{p}^{2}}{2}]{{\bf{E}}{\boldsymbol{^{\prime} }}}^{n}\\  &  & -[\frac{1}{{({\rm{\Delta }}t^{\prime} )}^{2}}-\frac{\gamma }{2{\rm{\Delta }}t^{\prime} }+\frac{{\omega }_{p}^{2}}{4}]{{\bf{E}}{\boldsymbol{^{\prime} }}}^{n-1}\}/[\frac{1}{{({\rm{\Delta }}t^{\prime} )}^{2}}+\frac{\gamma }{2{\rm{\Delta }}t^{\prime} }+\frac{{\omega }_{p}^{2}}{4}],\end{array}$$

The updating equation for **H**′ is in the same form as (13) by replacing **E**′, **D**′ and *ε*_0_, by **H**′, **B**′ and *μ*_0_, respectively, i.e.

14 $$\begin{array}{rcl}{{\bf{H}}{\boldsymbol{^{\prime} }}}^{n+1} & = & \{[\frac{1}{{\mu }_{0}{({\rm{\Delta }}t^{\prime} )}^{2}}+\frac{\gamma }{2{\mu }_{0}{\rm{\Delta }}t^{\prime} }]{{\bf{B}}{\boldsymbol{^{\prime} }}}^{n+1}-\frac{2}{{\mu }_{0}{({\rm{\Delta }}t^{\prime} )}^{2}}{{\bf{B}}{\boldsymbol{^{\prime} }}}^{n}\\  &  & +[\frac{1}{{\mu }_{0}{({\rm{\Delta }}t^{\prime} )}^{2}}-\frac{\gamma }{2{\mu }_{0}{\rm{\Delta }}t^{\prime} }]{{\bf{B}}{\boldsymbol{^{\prime} }}}^{n-1}+[\frac{2}{{({\rm{\Delta }}t^{\prime} )}^{2}}-\frac{{\omega }_{p}^{2}}{2}]{{\bf{H}}{\boldsymbol{^{\prime} }}}^{n}\\  &  & -[\frac{1}{{({\rm{\Delta }}t^{\prime} )}^{2}}-\frac{\gamma }{2{\rm{\Delta }}t^{\prime} }+\frac{{\omega }_{p}^{2}}{4}]{{\bf{H}}{\boldsymbol{^{\prime} }}}^{n-1}\}/[\frac{1}{{({\rm{\Delta }}t^{\prime} )}^{2}}+\frac{\gamma }{2{\rm{\Delta }}t^{\prime} }+\frac{{\omega }_{p}^{2}}{4}]\mathrm{.}\end{array}$$

Equations (8), (9), (13) and (14) form the FDTD updating equation set for LHMs in the *rest* frame. The field components in the *laboratory* frame can be calculated by solving Eqs (1) and (2) for **B**, **E**, and Eqs (3) and (4) for **D**, **H**, respectively at every time iteration.

## Results and Discussion

In our analysis, we model an LHM slab with its thickness equal to *L*. Its front and back interfaces are parallel and perpendicular to the *y*-direction, as shown in Fig. 1. Assume that the slab is moving at a constant speed along the *y*-direction, i.e. ${\bf{v}}=v\hat{y}$. To allow the LHM slab to be relatively static, the *rest* frame also moves at the same speed *v* along the *y*-direction. The reason for aligning the direction of movement with the coordinate axis is that the Lorentz transformation can be significantly simplified. For more general cases with arbitrary moving directions, the equations with additional terms can be derived in a similar manner.

### Implementation in one dimension and validation of the Lorentz-FDTD method

For the 1-D implementation of the Lorentz-FDTD method, plane-wave incidence is assumed, with only ${E}_{z}^{^{\prime} }$, ${D}_{z}^{^{\prime} }$, ${H}_{x}^{^{\prime} }$, and ${B}_{x}^{^{\prime} }$ components being non-zero. In the FDTD domain, the slab is infinite in both *x*- and *z*-directions. Under the 1-D assumption and ${\bf{v}}=v\hat{y}$, the Lorentz transformation equations (1–4) reduce to

15 $${B}_{x}^{^{\prime} }=\xi ({B}_{x}-\frac{v}{{c}^{2}}{E}_{z}),$$

16 $${E}_{z}^{^{\prime} }=\xi ({E}_{z}-v{B}_{x}),$$

17 $${D}_{z}^{^{\prime} }=\xi ({D}_{z}-\frac{v}{{c}^{2}}{H}_{x}),$$

18 $${H}_{x}^{^{\prime} }=\xi ({H}_{x}-v{D}_{z})\mathrm{.}$$

Solving for the field components in the *laboratory* frame gives

19 $${B}_{x}=\xi ({B}_{x}^{^{\prime} }+\frac{v}{{c}^{2}}{E}_{z}^{^{\prime} }),$$

20 $${E}_{z}=\xi ({E}_{z}^{^{\prime} }+v{B}_{x}^{^{\prime} }),$$

21 $${D}_{z}=\xi ({D}_{z}^{^{\prime} }+\frac{v}{{c}^{2}}{H}_{x}^{^{\prime} }),$$

22 $${H}_{x}=\xi ({H}_{x}^{^{\prime} }+v{D}_{z}^{^{\prime} })\mathrm{.}$$

Notice that the above equations can be also obtained simply by replacing primed field components by their unprimed ones and vice versa, and also letting *v* → −*v*, since the Lorentz transformation and its inverse transformation have the same form.

**Figure 2:**
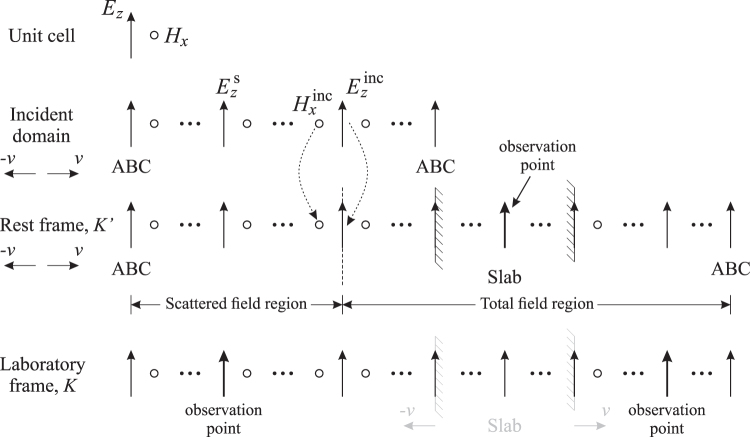
The 1-D FDTD simulation domain containing an incident domain for the implementation of the total-field scattered-field (TFSF) method, a *rest* frame, and a *laboratory* frame.

**Figure 3:**
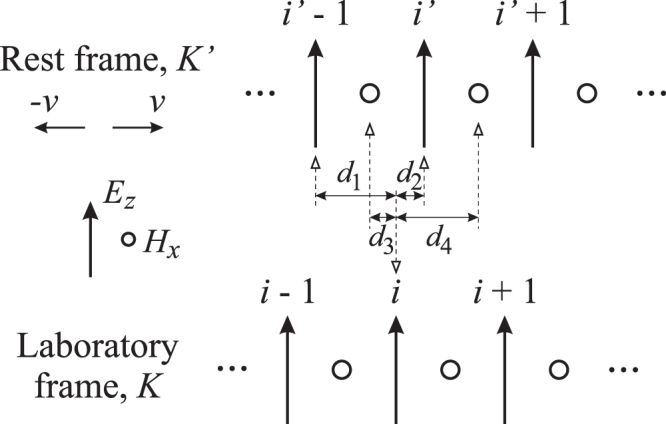
The grid layout in the FDTD domain. An offset is caused by the movement of the *rest* frame relative to the *laboratory* frame.

Figure 2 shows the 1-D FDTD simulation domains for the *rest* and *laboratory* frames, as well as an incident-field domain for the implementation of the total-field scattered-field (TFSF) method. The TFSF method is used to introduce the incident wave at the boundary between the scattered and total field regions (see Fig. 2), and only allows the wave to propagate toward the right side of the domain. The excitation source is introduced in the incident domain, and the arrows with dotted lines indicate that the ${E}_{z}^{{\rm{inc}}}$ and ${H}_{x}^{{\rm{inc}}}$ fields (calculated from FDTD simulations) are then introduced into the *rest* frame. Absorbing boundary conditions (ABCs) are applied to terminate the FDTD domain for both the incident domain and the *rest* frame. However for the *laboratory* frame, no ABCs are required and all field values are directly calculated by Eqs (19–22) at every FDTD time step. Two observation points are located in the *laboratory* frame which are assumed to be static. The distances from the left observation point to the TFSF boundary, and from the right boundary of the slab to the right observation point are both sufficiently long considering the cases of the slab moving in both directions, to ensure that only the scattered (reflected) and/or transmitted fields are recorded. The incident domain and the *rest* frame move at the same constant velocity, and due to such movement, a mapping of FDTD grids between the *rest* and *laboratory* frames is necessary when applying Eqs (19–22). Figure 3 shows the grid offset caused by the movement. In order to ensure accuracy in FDTD simulations, the field components in the *laboratory* frame are calculated using weighted average of adjacent components in the *rest* frame. For example, when calculating the *E*_*z*_ component, the following equation is used,

23 $${E}_{z}(i)=\xi \{[\frac{{d}_{1}}{{\rm{\Delta }}x}{E}_{z}^{^{\prime} }(i^{\prime} )+\frac{{d}_{2}}{{\rm{\Delta }}x}{E}_{z}^{^{\prime} }(i^{\prime} -\mathrm{1)}]+v[\frac{{d}_{3}}{{\rm{\Delta }}x}{B}_{x}^{^{\prime} }(i^{\prime} )+\frac{{d}_{4}}{{\rm{\Delta }}x}{B}_{x}^{^{\prime} }(i^{\prime} -\mathrm{1)}]\},$$

where *d*_1_, *d*_2_, *d*_3_, and *d*_4_ are the offset distances between the *E*_*z*_ component at location *i* in the *laboratory* frame, to two nearest ${E}_{z}^{^{\prime} }$ components at locations *i*′ and *i*′ − 1 in the *rest* frame, and two nearest ${B}_{x}^{^{\prime} }$ components at locations *i*′ and *i*′ − 1 in the *rest* frame, as defined in Fig. 3. Notice that *d*_1_ + *d*_2_ = *d*_3_ + *d*_4_ = Δ*x*, *d*_1_ + *d*_4_ = 1.5Δ*x*, and *d*_2_ + *d*_3_ = 0.5Δ*x*. The equations to calculate other field components in the *laboratory* frame can be derived similarly.

In addition to the field updating equations in both *rest* and *laboratory* frames introduced in the previous section, the source excitation also needs a special treatment. In our analysis, we assume that the metamaterial is moving while the source remains static with respect to the *laboratory* frame, thus the Lorentz transformation is applied to the excitation function to obtain its form in the *rest* frame (assuming sinusoidal excitation applied to the *E*_*z*_ component):

24 $${E}_{z}={E}_{0}\,\sin \,(2\pi fn{\rm{\Delta }}t),$$

25 $${E}_{z}^{^{\prime} }={E}_{0}\sqrt{\frac{1-\beta }{1+\beta }}\,\sin \,(2\pi f\sqrt{\frac{1-\beta }{1+\beta }}n{\rm{\Delta }}t^{\prime} ),$$

where *β* = *v*/*c*, and *f* is the frequency of the sinusoidal wave. It is evident from these equations that the incident wave in the *rest* frame undergoes a Doppler shift in both amplitude and frequency due to the movement of the *rest* frame.

In order to validate the Lorentz-FDTD method, we first model the slab as a perfectly electric conductor (PEC), and compare the calculated frequency shift and amplitude variation due to the movement of the slab from FDTD simulations with the theoretical ones. Here both cases of the slab moving away from the source $({\bf{v}}=+\,v\hat{y})$ and moving toward the source $({\bf{v}}=-\,v\hat{y})$ are considered. The source is located at the origin of the *rest* frame, *O*′, and the observation point is located on the −*y* axis of the *laboratory* frame to allow only the reflected fields from the PEC slab to be recorded. The operating frequency of the incident wave is *f* = 1 GHz. The FDTD cell size in the *rest* frame is Δ*y*′ = 5.0 × 10^−3^ m, equivalent to *λ*/60 where *λ* is the wavelength at the operating frequency. According to the Courant–Friedrichs–Lewy (CFL) condition^13^, the discretized time step is chosen as Δ*t*′ = Δ*y*′/*c* where *c* is the speed of light in free space.

**Figure 4:**
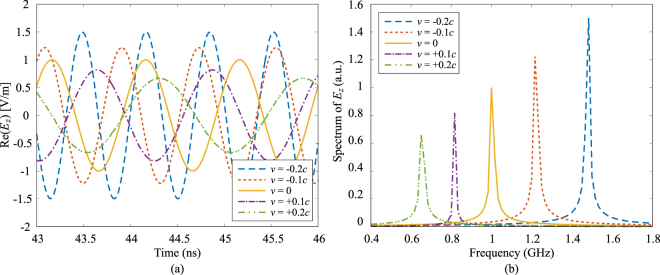
Waveforms of reflected electric field in (**a**) time and (**b**) frequency domains when the slab is modelled as a PEC and moving at various velocities. The frequency of incident wave is 1 GHz. A Doppler shift in both amplitude and frequency is clearly shown.

Figure 4 shows the reflected waveforms of electric field from the PEC when it is moving at various velocities both toward and away from the source.

It can be seen that both the amplitude and frequency of the reflected wave increase when the PEC slab moves toward the source (*v* < 0), and decrease when the slab moves away (*v* > 0). The theoretical Doppler shifts in amplitude and frequency can be calculated as^20^

26 $${E}_{0,r}=(\frac{1-v/c}{1+v/c}){E}_{0,i},\,\,{f}_{r}=(\frac{1-v/c}{1+v/c}){f}_{i},$$

where *E*_0,*i*_ and *f*_*i*_ are amplitude and frequency of the incident wave, and *E*_0,*r*_ and *f*_*r*_ are amplitude and frequency of the reflected wave. The comparison of calculated numerical values from our simulations are in excellent agreement with the theoretical ones calculated from Eq. (26) with a maximum error of less than 0.5%, which validates our Lorentz-FDTD method.

**Figure 5:**
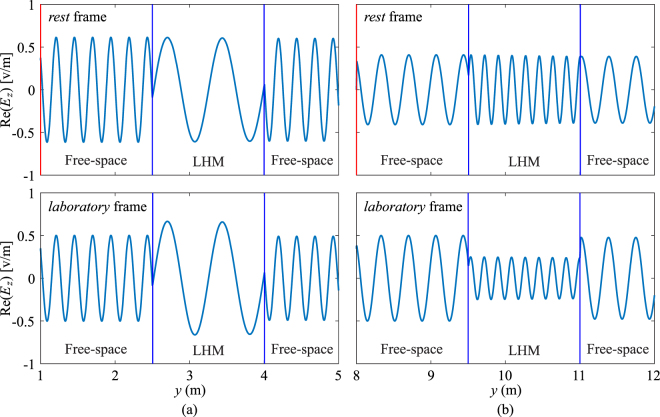
Distributions of electric field for (**a**) *v* = −0.2*c*, (**b**) *v* = +0.2*c* in *rest* and *laboratory* frames. Only a section of each frame is shown. The red vertical lines indicate the location of source. Notice the difference between the wave amplitude inside the LHM slab in *rest* and *laboratory* frames when the slab is moving toward or away from the source.

The slab is then modelled as an LHM with its thickness equal to 1.5 m. The spatial distributions of electric field in both *rest* and *laboratory* frames are plotted and shown in Fig. 5 for two cases of *v* = −0.2*c* and *v* = +0.2*c*.

**Figure 6:**
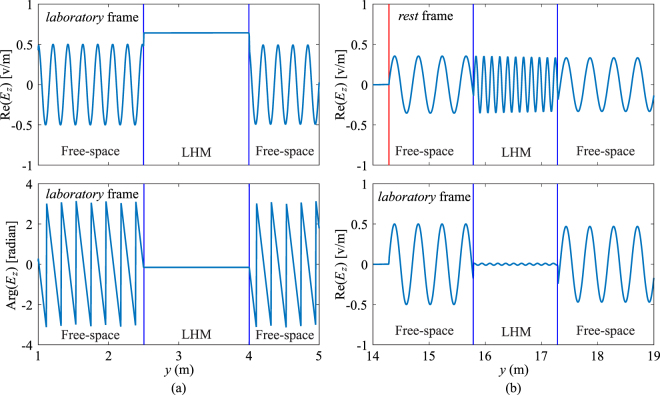
Distributions of electric field for (**a**) *v* = −0.3322*c* and (**b**) *v* = +0.3322*c*. Only a section of each frame is shown. The red vertical line indicates the location of source. When the slab is moving toward the source at the critical velocity, a constant amplitude of wave and zero spatial phase delay are observed inside the slab. When the slab is moving away from the source at the critical velocity, the amplitude of wave retains a very small value inside the slab.

The electric field distributions directly observed in the *rest* frame shows that the amplitude remains the same after the wave enters the LHM slab, independent of the slab’s moving direction, while the spatial frequency decreases or increases when the slab moves toward or away from the source, respectively. In the *laboratory* frame, on the other hand, inside the LHM slab the spatial frequency remains the same as that in the *rest* frame, while the wave amplitude increases for the case of *v* < 0 and decreases when *v* > 0. By varying the velocity in simulations, some interesting wave behaviours can be observed. Particularly, when the slab moves toward the source and the velocity gradually increases, backward waves are generated inside the LHM slab. When the velocity is further increased after passing a critical value, backward waves change to forward waves which cannot be observed inside a static LHM slab. In simulations, this critical velocity is found to be *v* ≈ −0.3322*c*. In addition, the spatial frequency of wave also decreases when the velocity gradually increases, and the spatial frequency increases again after this critical velocity. At the exact critical velocity, we can observe a wave with spatially constant amplitude inside the LHM slab. In other words, waves experience zero spatial phase delay through the LHM slab, as shown in Fig. 6(a). The constant wave amplitude and zero phase delay inside the LHM slab can be explained by the effective permittivity and permeability of the slab as follows. According to the Lorentz transformation for the angular frequency, *ω*′^20^,

27 $$\omega ^{\prime} =\xi (\omega -{\bf{v}}\cdot {\bf{k}}),$$

where *ω* and **k** are the angular frequency and wave vector in the *laboratory* frame, respectively, when the LHM slab is moving at various velocities, the effective permittivity and permeability in Eq. (5) vary accordingly. Theoretically, when the slab is moving at *v* = −*c*/3, both effective permittivity and permeability are equal to zero, resulting in a slab of zero-index material which introduces zero phase delay to the incident wave^26^. Nonetheless, the slight discrepancy (with an error of 0.3%) between the theoretical velocity (*v* = −*c*/3) and the numerical value *v* = −0.3322*c* obtained from FDTD simulations is mainly due to the numerical dispersion effect, and can be further reduced when a finer FDTD mesh is used. However, this may result in an excessive requirement for the computational time and computer memory. Note that the plasma frequency remains unchanged regardless of the moving velocity of the LHM slab^27^.

If the LHM slab moves away from the source, it is observed in simulations that, the waves inside the LHM are always backward waves. However, in the *laboratory* frame the amplitude of wave inside LHM varies with the velocity. It is found that there also exists a critical velocity with the same absolute value, i.e. *v* ≈ +0.3322*c* below which the wave amplitude decreases when the velocity increases, and the amplitude increases when *v* is increased further from the critical value. At this critical velocity, the amplitude of waves inside the LHM slab reaches its minimum value, as shown in Fig. 6(b). Similar to the analysis above for *v* < 0, the minimum amplitude inside the LHM slab in the laboratory frame can be also explained by the effective permittivity and permeability. Applying Maxwell’s equations inside the LHM slab in the *rest* frame, ${B}_{x}^{^{\prime} }=({k}_{y^{\prime} }/\omega ^{\prime} ){E}_{z}^{^{\prime} }$, the 1-D Lorentz transformation for the *E*_*z*_ component, Eq. (20) can be written as,

28 $${E}_{z}=\xi (1+v\frac{{k}_{y^{\prime} }}{\omega ^{\prime} }){E}_{z}^{^{\prime} },$$

where ${k}_{y^{\prime} }=-\,(\omega ^{\prime} /c)\sqrt{{\varepsilon }_{r}(\omega ^{\prime} ){\mu }_{r}(\omega ^{\prime} )}$ (due to backward waves). At the exact theoretical velocity, *v* = +*c*/3, according to Eqs (5) and (27), the effective material parameters due to the movement of the LHM slab can be calculated as *ε*_*r*_ = *μ*_*r*_ = −3. Substituting the above values into Eq. (28) results in *E*_*z*_ = 0, i.e. zero field amplitude inside the LHM slab moving at *v* = +*c*/3. Again the slight discrepancy between the theoretical value of *v* = +*c*/3 and *v* = +0.3322*c* obtained in FDTD simulations is mainly due to the numerical dispersion effect. Note that the above results are obtained when a small amount of loss is used for the LHM (*ε*_*r*_ = *μ*_*r*_ = −1 − 0.001*j*). When the loss increases, the behaviors of waves remain the same, while the amplitude of wave decreases with propagation distance.

**Figure 7:**
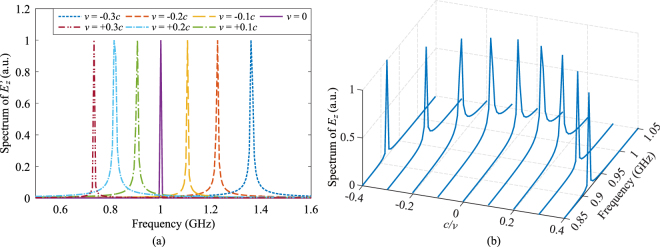
Normalised spectra of recorded electric field when the observation is located (**a**) inside the LHM slab in the *rest* frame, and (**b**) behind the LHM slab in the *laboratory* frame. Frequency shifts are observed when the observation point is located within the slab (moving at the same speed). Behind the LHM slab, a red-shift of wave frequency is observed regardless of the slab’s moving direction.

To investigate the frequency domain characteristics of transmitted waves, we record the fields both inside the LHM slab in the *rest* frame and in the free space behind the LHM slab in the *laboratory* frame. As shown in Fig. 7, due to the movement of the slab, the spectra of waves observed inside the LHM slab shift to different frequencies, which is a similar behavior to dielectric slabs.

However, behind the LHM slab the transmitted waves experience a red-shift of frequency when the slab moves in either direction, as shown in Fig. 7(b).

The above results show significantly different behaviours of transmitted waves and waves inside a moving LHM slab for the case of normal incidence. For the oblique incidence, it is well known that negative refractions occur at the interfaces between the free space and LHM slab. Using our proposed Lorentz FDTD method implemented in 2-D, we also investigate the effect when the LHM moves at a high speed.

### Two-dimensional implementation

In the 2-D implementation, we consider the transverse electric (TE*z*) mode with ${E}_{x}^{^{\prime} }$, ${D}_{x}^{^{\prime} }$, ${E}_{y}^{^{\prime} }$, ${D}_{y}^{^{\prime} }$, ${H}_{z}^{^{\prime} }$, and ${B}_{z}^{^{\prime} }$ components being non-zero. Same as the 1-D case, the slab is moving along the *y*-direction, i.e. ${\bf{v}}=\pm \,v\hat{y}$. The Lorentz transformation equations from the *rest* to *laboratory* frames can be derived as

29 $${B}_{z}=\xi ({B}_{z}^{^{\prime} }-\frac{v}{{c}^{2}}{E}_{x}^{^{\prime} }),$$

30 $${E}_{x}=\xi ({E}_{x}^{^{\prime} }-v{B}_{z}^{^{\prime} }),$$

31 $${E}_{y}={E}_{y}^{^{\prime} }/\xi ,$$

32 $${D}_{x}=\xi ({D}_{x}^{^{\prime} }-\frac{v}{{c}^{2}}{H}_{z}^{^{\prime} }),$$

33 $${D}_{y}={D}_{y}^{^{\prime} }/\xi ,$$

34 $${H}_{z}=\xi ({H}_{z}^{^{\prime} }-v{D}_{x}^{^{\prime} }).$$

**Figure 8:**
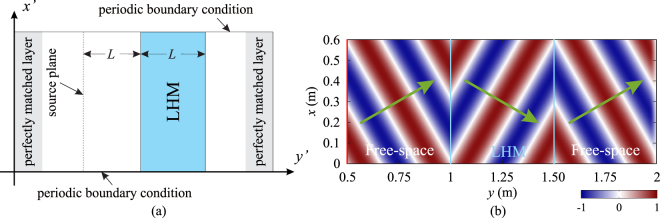
(**a**) The 2-D FDTD simulation domain for modelling oblique incidence on the LHM slab. (**b**) Distribution of magnetic field component, *H*_*z*_ demonstrating negative refraction at the interfaces between the free space and LHM slab. The angle of incidence is 30 degrees.

Figure 8(a) shows the 2-D FDTD simulation domain in the *rest* frame. Perfectly matched layers (PMLs)^28^ are used to terminate the domain in *y*-direction, and periodic boundary conditions (PBCs) are applied in *x*-direction to model an infinitely-long LHM slab. The thickness of the LHM slab is *L* = 0.5 m, and the source plane is located at a distance equal to the thickness of LHM slab, as shown in Fig. 8(a). The line source can be defined as

35 $${H}_{z}^{^{\prime} }(i^{\prime} ,{j}_{s}^{^{\prime} })={H}_{z}^{^{\prime} }(i^{\prime} ,{j}_{s}^{^{\prime} })+{H}_{0}\sqrt{\frac{1-\beta }{1+\beta }}\,\sin \,(2\pi f\sqrt{\frac{1-\beta }{1+\beta }}n{\rm{\Delta }}t^{\prime} ){e}^{-j{k}_{x^{\prime} }i^{\prime} {\rm{\Delta }}x^{\prime} },$$

where ${j}_{s}^{^{\prime} }$ is the location of source along *y*′-direction, *i*′ ∈ [1, *I*′] is the index of cell location, *I*′ is the total number of cells in *x*′-direction, and *k*_*x*′_ is the *x*′-component of the wave vector, and can be calculated as

36 $${k}_{x^{\prime} }={k}_{0}(1-\,\frac{v}{c}\,\cos \,{\theta }_{i})\,\sin \,{\theta }_{i},$$

where *k*_0_ is the free space wave number, and *θ*_*i*_ is the angle of incidence. Note that a correction term, (*v*/*c*)cos*θ*_*i*_ is included in Eq. (36) due to the movement of the slab. The operating frequency is 1 GHz, same as in 1-D FDTD simulations in the previous subsection. The FDTD cell size also remains the same, i.e. Δ*x*′ = Δ*y*′ = 5.0 × 10^−3^ m (*λ*/60). The time step is chosen according to the CFL condition, ${\rm{\Delta }}t^{\prime} ={\rm{\Delta }}x^{\prime} /\sqrt{2}c$^13^. The TFSF method is implemented for the 2-D case for the *rest* frame. For the *laboratory* frame, the fields are directly calculated by Eqs (29–34) and no boundary conditions are required. In all cases of oblique incidence, we restrict the angle of incidence to be 30 degrees. As it is clearly shown in Fig. 8(b), when the LHM slab is static, negative refractions occur at the interfaces between the free space and LHM slab.

**Figure 9:**
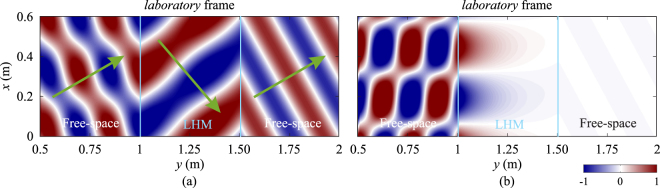
Distributions of magnetic field component, *H*_*z*_ in the *laboratory* frame when the LHM slab is moving toward the source at two different velocities: (**a**) *v* = −0.1*c*, (**b**) *v* = −0.2*c*. In both cases, the angle of incidence is 30 degrees.

However, when the slab is non-static, the field distributions appear to be very different, especially when the moving velocity is high. Figure 9 shows the distributions of magnetic field component, *H*_*z*_ in the *laboratory* frame when the LHM slab is moving toward the line source with two different moving speeds, *v* = −0.1*c* and *v* = −0.2*c*. It can be observed from Fig. 9(a) that when *v* < 0, the LHM slab is no longer matched to the free space and reflections occur at the interfaces. The angle of refraction also becomes larger than the angle of incidence (in the case of static LHM, these two angles are equal), and fields inside the LHM slab have a larger wavelength comparing with the free-space one. When the velocity is further increased from *v* = −0.1*c*, the LHM slab becomes more reflective, and more components of waves inside the LHM slab turn into evanescent such that little or no fields can be transmitted through the slab when the velocity is high, as shown in Fig. 9(b). Note that the field distributions in the *rest* frame are very similar to those shown in Fig. 9, thus only distributions in the *laboratory* frame are shown.

**Figure 10:**
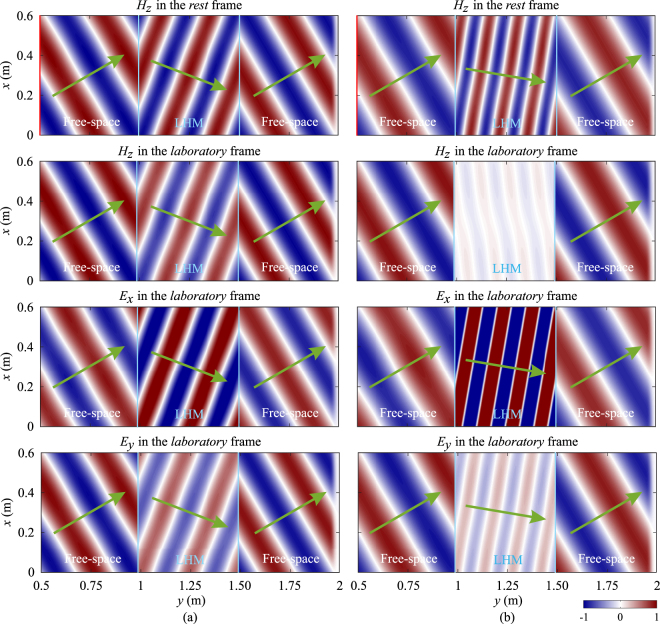
Distributions of field components, *H*_*z*_, *E*_*x*_ and *E*_*y*_ in the *rest* and *laboratory* frames when the LHM slab is moving away from the source at two different velocities: (**a**) *v* = +0.1*c*, (**b**) *v* = +0.3322*c*. In both cases, the angle of incidence is 30 degrees. The line source is indicated by the red vertical lines.

When the LHM slab is moving away from the line source, we can observe a smaller angle of refraction and a smaller wavelength inside the slab. Besides, the LHM slab is still matched to the free space and no apparent reflections occur at the interfaces, as shown in Fig. 10(a) for the case of *v* = +0.1*c*. When the velocity increases, both the angle of refraction and the wavelength of fields inside the LHM slab become even smaller. Moreover, it can be also observed that as the velocity gradually increases, the magnitudes of *H*_*z*_ and *E*_*y*_ components inside the LHM slab decrease, while the magnitude of *E*_*x*_ component increases. When the LHM slab moves at the critical velocity of *v* = +0.3322*c*, the *H*_*z*_ component inside the slab retains a very small magnitude, while the magnitude of *E*_*x*_ becomes very high, as shown in Fig. 10(b). After passing through the LHM slab, the magnitudes of *H*_*z*_, *E*_*x*_ and *E*_*y*_ are restored to their original values before the LHM slab with slight decay due to the loss in the slab, and the magnitudes decrease as the amount of loss increases. Thus we can conclude that when the LHM slab moves away from the source at the critical velocity, it functions as a field converter.

## Conclusion

In conclusion, we have applied a relativistic FDTD method by combining the Lorentz transformation with an ADE dispersive FDTD method for analysing metamaterials moving at a high speed. As an example, we consider the left-handed metamaterial (LHM) with both relative permittivity and permeability equal to −1. It is shown from our results that when the LHM slab is moving toward a source, the spatial frequency inside the LHM slab decreases, and the spatial frequency increases when the slab is moving away from the source. For the case of normal incidence, it is also found that there exists a special velocity at which fields inside the LHM slab experience a zero spatial phase delay when the slab is moving toward the source; when the slab is moving away, the magnitudes of both electric and magnetic fields inside the slab reach their minimum values. For the case of oblique incidence, the angle of refraction is inverse proportional to the moving velocity, and the fields inside the LHM slab become evanescent when the slab is moving toward the source above a certain velocity; when the slab is moving away, it functions as a field converter and the maximum conversion is achieved when the slab moves at the critical velocity. In the present work we have considered the LHM as an example, and results show considerably different wave behaviours comparing with the static case. Using our proposed Lorentz-FDTD method, other types of metamaterials can be readily analysed such as the mu-negative material (MNG), zero-index material and metasurfaces etc. We anticipate that when these metamaterials move at high velocities, some interesting properties can be further discovered.
